# A Hypomorphic Mutation Reveals a Stringent Requirement for the ATM Checkpoint Protein in Telomere Protection During Early Cell Division in *Drosophila*

**DOI:** 10.1534/g3.113.006312

**Published:** 2013-06-01

**Authors:** Patrizia Morciano, Yi Zhang, Giovanni Cenci, Yikang S. Rong

**Affiliations:** * Laboratory of Biochemistry and Molecular Biology, National Cancer Institute, National Institutes of Health, Bethesda, Maryland 20892; † Sapienza, University of Rome, 00185 Rome, Italy

**Keywords:** ATM kinase, Drosophila hypomorphic mutation, MRN complex, maternal lethal, telomere protection

## Abstract

Using *Drosophila* as a model system, we identified a stringent requirement for the conserved function of Ataxia Telangiectasia Mutated (ATM) in telomere protection during early embryonic development. Animals homozygous for a hypomorphic mutation in *atm* develop normally with minimal telomere dysfunction. However, mutant females produce inviable embryos that succumb to mitotic failure caused by covalent fusions of telomeric DNA. Interestingly, although the *atm* mutation encodes a premature stop codon, it must not have eliminated the production of the mutant protein, and the mutant protein retains kinase activity upon DNA damage. Moreover, although the embryonic phenotype of this mutation resembles that of hypomorphic mutations in the MRN complex, the function of MRN appears normal in the *atm* embryos. In contrast, there is a prominent reduction of the level of HipHop, an essential member of the *Drosophila* capping complex. How ATM functions in telomere protection remains poorly understood. The amenability of *Drosophila* embryos to molecular and biochemical investigations ensures that this newly identified mutation will facilitate future studies of ATM in telomere maintenance.

The Ataxia Telangiectasia Mutated (ATM) protein is best known for its role in sensing and repairing DNA damage (reviewed in Lee and Paull 2007). However, ATM is also important for telomere maintenance. The yeast ATM homolog Tel1 was first discovered for its role in maintaining telomere length (Lustig and Petes 1986). Although the function of ATM at telomeres is conserved through evolution, the underlying mechanism is not well understood. It has been suggested that the fundamental role of ATM at telomeres is in end processing, similar to its function at DSBs, but independent of the telomerase function (Ritchie *et al.* 1999; Gao *et al.* 2010a). This finding is consistent with results from ATM studies in *Drosophila*, which use telomerase-independent mechanisms to maintain the essential functions of telomeres (reviewed in Rong 2008a).

We along with others have shown that the ATM protein, which is encoded by the *telomere fusion* (*tefu*) gene in *Drosophila*, is essential for preventing telomere fusion in proliferating tissues (Bi *et al.* 2004; Oikemus *et al.* 2004; Silva *et al.* 2004; Song *et al.* 2004). In addition, ATM functions in the same telomere-protecting pathway as the conserved Mre11-Rad50-Nbs (MRN) complex, which is partially redundant with the pathway controlled by the ATM-related ATR protein (Bi *et al.* 2005; Ciapponi *et al.* 2006; Oikemus *et al.* 2006). Moreover, this pathway relationship is conserved in yeast (Moser *et al.* 2009) and quite possibly in plants (Vespa *et al.* 2005) and higher eukaryotes.

One possible function of ATM at *Drosophila* telomeres is to facilitate the recruitment of capping proteins. Although the binding of the HOAP capping protein to telomeres appears normal in *atm* mutants, as assayed by immunostaining of mitotic chromosomes (Bi *et al.* 2004; Rong 2008b), loading of HP1/Orc-associated Protein (HOAP) to *atm*-mutant telomeres might be defective after all, as binding can be further eroded by the loss of ATR. Although the loss of ATR alone does not impair telomere protection, it abolishes HOAP loading to telomeres in the *atm* mutant and exacerbates telomere uncapping in the *atm atr* double mutant to a degree similar to that in the single *caravaggio* (*cav*) mutant that encodes a defective HOAP protein (Cenci *et al.* 2003; Bi *et al.* 2005; Rong 2008b). In addition, the level of the HipHop capping protein and its localization to telomeres are moderately reduced in an *atm* mutant (Gao *et al.* 2010b), consistent with the fact that telomeres in *atm* mutants have a suboptimal level of protection.

Recently, we discovered a stringent requirement for the MRN complex in telomere protection during early development (Gao *et al.* 2009). Hypomorphic mutations of *mre11* and *nbs* support viability, but mutant females are unable to produce viable progeny due to rampant mitotic failure during the earliest cell cycles in embryos. Here, we characterized a hypomorphic mutation in *Drosophila atm* that has a similar maternal lethal phenotype. We show that loss of maternal ATM function leads to telomere fusion in the embryos. At the cellular level, this hypomorphic mutation displays features of telomere dysfunction similar to those caused by severe loss of function mutations of the capping machinery. This mutation will facilitate the future elucidation of telomere protection mechanisms by ATM, particularly due to the amenability of *Drosophila* embryos to molecular and biochemical investigations.

## Materials and Methods

### *Drosophila* stocks and genetics

The *tefu^ZIII-5190^* stock and its corresponding parental stock were obtained from the Zuker Collection (Koundakjian *et al.* 2004). The *tefu^stg^* allele has been described previously (Gong *et al.* 2005). The *atm^6^* allele was obtained from the Bloomington stock center. The stock that contains the *atm^+^* transgene was provided by Dr. Shigla Campbell at the University of Alberta. The *mre11^35K1^* and *nbs^1^* single mutants, as well as the *atm atr* double mutant have been described previously (Bi *et al.* 2004; Bi *et al.* 2005).

To measure the effect of *tefu^ZIII-5190^* on viability, heterozygous stocks of *tefu^ZIII-5190^* and *tefu^stg^*, each balanced over the TM6B chromosome, were crossed. Progeny were scored as heterozygous or transheterozygous for *tefu*. The expected ratio between these two classes is 2:1. The observed ratio was 1.4:1 (n = 797). The transheterozygous females were mated with their wild-type siblings to produced the embryos used in phenotypic analyses.

### Molecular biology

Between 0.2 and 1 μg of genomic DNA from embryos was used in a 50- to 100-μL polymerase chain reaction (PCR) to recover telomere fusion junction essentially as previously described (Gao *et al.* 2009). Five primers were designed from the *orf* region of the HeT-A element (Supporting Information, Table S1). The combinations: 1 + 4, 2 + 4, 3 + 4, and 3 + 5 were used for junction isolation. PCR products were cloned using TOPO TA cloning and subsequently sequenced.

Genomic DNA from *tefu^ZIII-5190^* homozygotes and its parental stock were used as the template in PCRs to amplify 13 fragments covering the *tefu* genomic region. PCR products were cloned using TOPO TA cloning, and independent clones of a particular fragment were sequenced to identify nucleotide differences between the two stocks. The primers are listed in Table S1.

### Cytology and Western blotting

Mitotic chromosome preparations from larvae and embryos were made as previously described (Gao *et al.* 2009). 4′,6-diamidino-2-phenylindole (DAPI) and antibody staining of embryos were performed as described previously (Gao *et al.* 2009). Embryo extracts were obtained from embryos as previously described (Gao *et al.* 2010b). Antibodies against HipHop and HOAP were described previously (Gao *et al.* 2010b). The antibodies against MRN have been previously described (Gao *et al.* 2009). The anti-Giotto antibody (Giansanti *et al.* 2006) was provided by M. Giansanti (SAPIENZA, University of Rome).

To detect damage-induced phosphorylation of H2AvD, larvae were irradiated with 1000 rads. At the indicated time points, proliferating tissues (brains and imaginal discs) were dissected and extracts were generated. A polyclonal antibody raised against a phosphorylated peptide from H2AvD was used to detect H2AvD and its phosphorylated form as previously described (Madigan *et al.* 2002). Due to depletion of this antibody stock, we later used an affinity purified rabbit antibody against the phosphorylated form of H2AvD (Rockland Inc., Gilbertsville, PA).

## Results and Discussion

### A hypomorphic mutant of *atm/tefu* causes maternal lethality

In a genetic screen for cell cycle mutants, Rickmyre *et al.* (2007) identified the ZIII-5190 stock from the Zuker collection as harboring a potential mutation in the *tefu* gene by the fact that ZIII-5190 failed to complement a small chromosomal deletion for female fertility, which eliminates part of *tefu*. We repeated the complementation test with our *tefu^stg^* allele that specifically affects *tefu* (Bi *et al.* 2004). As we showed before, the *tefu^stg^* allele causes pupal lethality as cells in proliferating tissues suffer genome instability caused by telomere fusion. In contrast, we recovered *tefu^ZIII-5190^/tefu^stg^ trans*-heterozygotes at a Mendelian ratio (see *Materials and Methods*), indicating that the *tefu^ZIII-5190^* allele minimally affects viability. Consistently, a near background level of telomere fusion, as measured by examining mitotic chromosome preparations (average 0.06 fusion, n = 337), was found in *tefu^ZIII-5190^* larval neuroblasts, which is significantly lower than the average of three fusions reported earlier for *tefu^stg^* neuroblasts (Bi *et al.* 2004).

**Figure 1:**
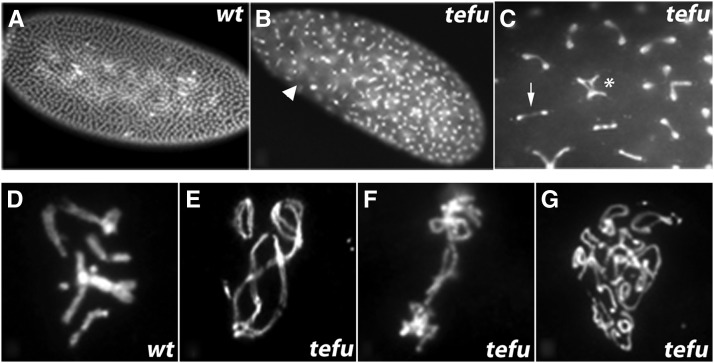
Mitotic defects in *m-tefu^5190^* embryos. (A−B) DAPI-staining of 0−2 hr wild-type (*wt*) and *m-tefu^5190^* (*tefu*) embryos. Although *wt* embryos show a uniform distribution of nuclei on the cortex, *m-tefu^5190^* embryos exhibit an aberrant pattern and the presence of large nuclei-free areas (arrowhead). (C) A close-up of panel B. Nuclei connected by chromatin bridges (arrow) and multilobed nuclei (asterisk) are abundant in *m*-tefu*^5190^* embryos. (D−G) Mitotic chromosome preparations from *wt* and *m-tefu^5190^* (*tefu*) embryos. Whereas all wild-type chromosomes are individually distinct (D), metaphase chromosomes from *m*-tefu*^5190^* exhibit telomeric fusions that give rise to chromosome chains (E), anaphase bridges (F), and hyperploid cells (G).

Females that are homozygous for *tefu^ZIII-5190^* or *trans*-heterozygous for *tefu^ZIII-5190^* and *tefu^stg^* produced a normal amount of eggs; however, none of more than 10,000 embryos counted hatched regardless of the genotypes of the mated males. DAPI-staining revealed significant development of these embryos (Figure 1), indicating that the embryos were fertilized and embryonic lethality was caused by the defective maternal contribution from the *tefu* mutant allele. The *tefu^ZIII-5190^* allele also failed to complement another pupal lethal allele, *atm^6^* (Silva *et al.* 2004). Heterozygous *tefu^ZIII-5190^*/*atm^6^* females also did not produce viable offspring, but this defect could be rescued by a wild-type *tefu* transgene. Taken collectively, our genetic analyses strongly suggest that the *tefu^ZIII-5190^* allele is a partial loss-of-function allele of *tefu* that causes maternal effect lethality.

### Telomere fusion causes mitotic failure in embryos from mutant females

We observed nuclear patterns indicative of mitotic failure in DAPI-stained embryos produced by females that are *tefu^ZIII-5190^*/*tefu^stg^*, hereafter referred to as *maternal-tefu^ZIII-5190^*/*tefu^stg^* (*m-tefu^5190^*) embryos (Figure 1, B and C). First, we observed chromosome bridges between segregating nuclei. Second, we observed multilobed nuclei, possibly resulting from a second round of mitosis after failed chromosome segregation. Finally, we observed large areas in the embryos that are free of nuclei, indicating cell-cycle defects due to “sinking” of abnormal nuclei to the interior after exiting the cell cycle, leaving nuclear-free areas at the surface (Raff and Glover 1988).

ATM prevents telomere fusion in the dividing tissues of larvae; therefore, we hypothesized that the mitotic failure observed in *m-tefu^5190^* embryos could be due to telomere uncapping. By using a recently developed protocol for producing mitotic chromosome preparations from embryos (Gao *et al.* 2009), we obtained convincing cytological evidence that *m-tefu^5190^* embryos experience telomere fusions (Figure 1, E−G).

**Figure 2:**
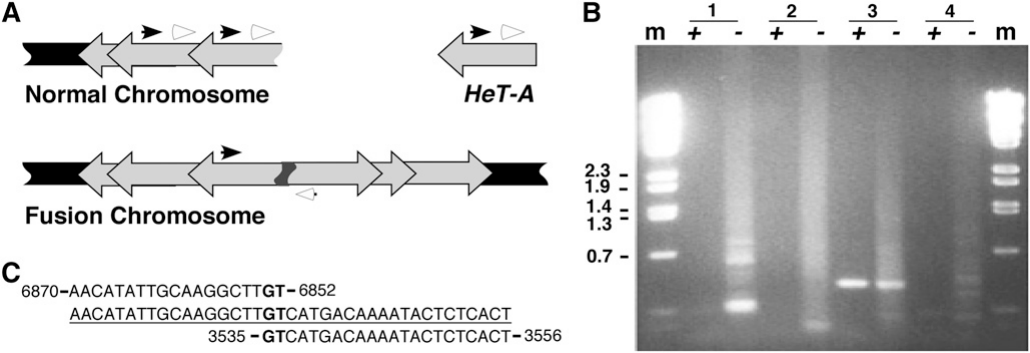
Telomere fusions in *m*-tefu*^5190^* are covalent DNA linkages. (A) Schematic of the PCR procedure used to recover telomere fusion junctions. Telomeric HeT-A retro-transposons are depicted as block arrows in gray. They attach to chromosomal DNA (in black) unidirectionally. Black and white arrowheads denote a pair of telomere-facing primers. They anneal to multiple positions along the HeT-A arrays. The top diagram denotes the wild-type situation in which the PCRs are not expected to be productive. The middle diagram depicts a telomere fusion in which PCR with some primer pairs will lead to productive amplifications. (B) A picture of a DNA gel electrophoresis showing PCR products obtained using wild type (*+*) or *m-tefu^5190^* (*−*) DNA templates. The primer combinations are listed at the top. m: marker DNA with sizes in kb. (C) Sequence of a fusion junction from *m-tefu^5190^*. The nucleotide numbers are from GenBank entry U06920.2. Three strands (in the 5′ to 3′ direction) are shown, with the actual sequences connected through a fusion (underlined). The rest of the sequences are those predicted from U06920.2. The top sequence is from a telomere that fused with another telomere (bottom sequence), giving rise to the fusion product denoted in the middle sequence. The fusion was created by the use of an overlapping “GT” (in bold) microhomology for repair.

To investigate whether the “fusions” observed cytologically indeed represent covalent attachment of chromosome ends, we used a recently developed PCR protocol to detect fusion junctions (Gao *et al.* 2009). This protocol is based on the fact that telomeric transposons in *Drosophila* are arranged as directed repeats such that the use of two transposon-derived primers oriented in the direction of the telomere is unlikely to support productive amplification of DNA samples from wild type flies. The same pair of primers, however, would amplify telomere fusion junctions on the DNA templates from uncapping mutants (Figure 2A). Using four different combinations of primer pairs (Table S1), we generated abundant PCR products of various sizes from *m-tefu^5190^* DNA, but not from wild type embryonic DNA (Figure 2B). Sequencing of 15 independent clones of potential fusion junctions identified signatures of nonhomologous end joining of telomeric transposons in a head-to-head fashion in all of the clones (one is shown in Figure 2C, the others in Table 1). In summary, our results indicate that maternal lethality of *m-tefu^5190^* embryos is caused by end-to-end fusions that impede chromosome segregation during cell division.

**Table 1:**
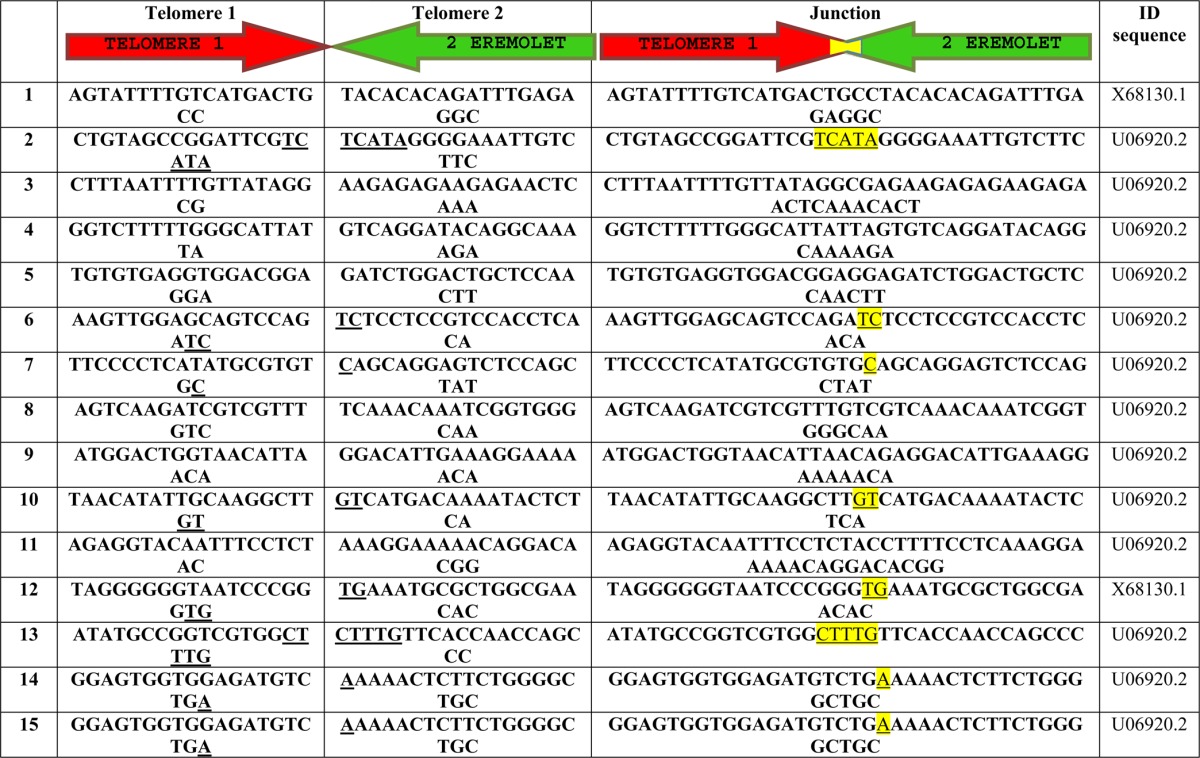
Telomere fusion junctions

Fifteen independent telomere fusion events were listed, with the sequences of the two “parental” telomeres listed as “Telomere 1” (red telomere) and “Telomere 2” (green telomere). For each telomere, the sequence denotes the strand that is going from centromere to telomere. In the “Junction” column, apparent microhomology used during NHEJ has been underlined, which include fusion events 2, 6, 7, 10, 12, 13, 14, 15. For events 3, 4, 8, 9, 11, “filler DNA” was used during NHEJ and the involved nucleotides are shown in black. “ID sequence” are Genbank numbers for the sequence used to deduced the fusion events.

### The *tefu^ZIII-5190^* mutation is a premature stop codon

We set out to identify the DNA lesion responsible for the mutation in *tefu^ZIII-5190^* to better understand the molecular mechanism underlying the uncapping phenotype. We amplified via PCR, cloned, and sequenced 13 overlapping fragments covering the entire *tefu* coding region and approximately 800 bp of the 5′ and 3′ UTR from *tefu^ZIII-5190^* homozygous and the parental wild-type stocks. We identified (1) a nonsense mutation (G to A) that changes a Tryptophan (TGG) at the predicted position of 356 to a STOP (TGA), (2) three synonymous changes, and (3) a single C to A change in one of the introns. We thought it most likely that the W356* change is responsible for the *tefu^ZIII-5190^* mutation. To further explore this possibility, we recovered a cDNA fragment by using primers that span the W356* mutation and a downstream intron to recover PCR products exclusively from mRNA but not from genomic DNA. Sequencing of this fragment identified the W356* mutation in *tefu^ZIII-5190^* but not in wild-type, suggesting that the mutation is part of the *tefu* transcript in the mutant.

The hypomorphic nature of *tefu^ZIII-5190^* seems incompatible with the nature of the mutation (a premature stop codon). In addition, the W54* mutation in the *atm^1^* allele causes a lethal allele but lies further to the N-terminus than W356* (Pedersen *et al.* 2010). The hypomorphic nature of W356* suggests that it does not result in the elimination of the ATM protein and that perhaps a downstream START codon is utilized. Currently available reagents did not allow us to further address this issue.

### The *tefu^ZIII-5190^* mutation does not disrupt MRN localization

**Figure 3:**
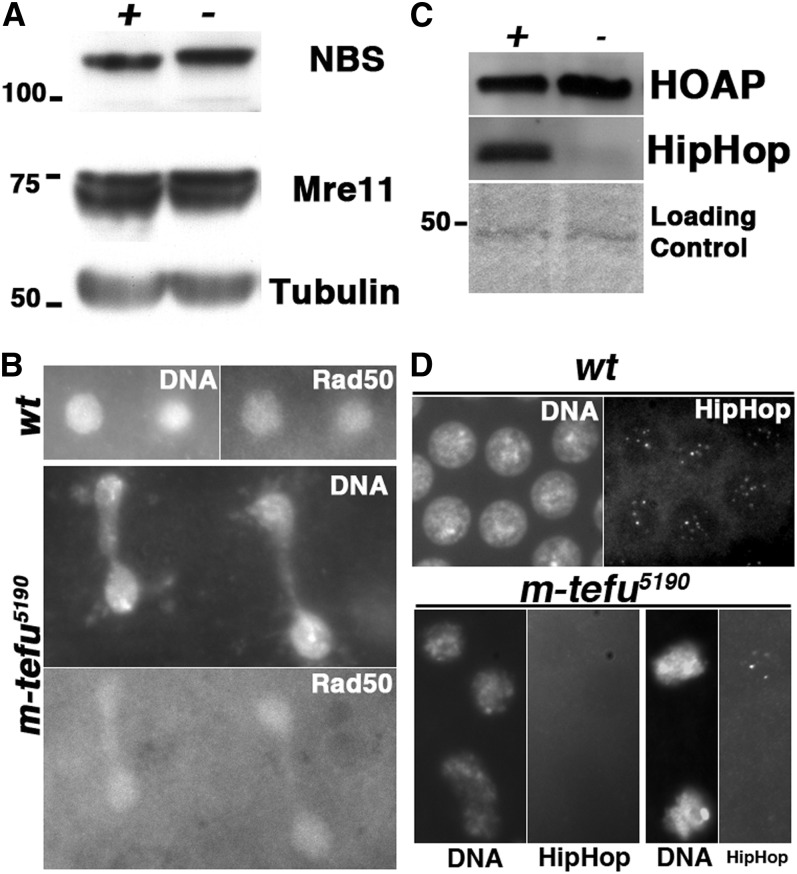
Integrity of the telomeric complexes in *m-tefu^5190^* embryos. (A) Western blot analysis of Nbs and Mre11 in wildtype (+) and *m-tefu^5190^* (−) embryonic extracts. Tubulin is probed as a loading control and molecular marker weights (kD) are indicated to the left. (B) Localization of Rad50 in *tefu^ZIII-5190^*. Gray scale pictures show DAPI-stained DNA or antibody-stained Rad50. (C) Western blot analysis of the levels of HipHop and HOAP in *m-tefu^5190^* (*−*) embryos. (D) Localization of HipHop in *tefu^ZIII-5190^*. Grayscale pictures show DAPI-stained DNA or antibody-stained HipHop. In wild-type, HipHop forms nuclear foci. In the *m-tefu^5190^* mutant, nuclei lacking of HipHop signals are shown on the left. On the right are two nuclei, one of which shows HipHop foci.

The cellular defects in *m-tefu^5190^* embryos phenocopy those in embryos produced by mothers with a hypomorphic mutation in either *mre11* or *nbs* (Gao *et al.* 2009). In those mutants, the nuclear localization of Mre11 and Rad50 is defective due to depletion of the maternal Nbs protein. Given the fact that ATM and MRN function in the same pathway to prevent telomere fusion in larval tissues, we investigated whether telomere uncapping in *m-tefu^5190^* embryos is caused by defective MRN function. We observed no obvious reduction in the total level of Mre11 or Nbs proteins in the mutant embryos (Figure 3A). Moreover, Rad50 localization to chromatin appears normal (Figure 3B). Therefore, the *tefu^ZIII-5190^* mutation does not seem to affect MRN function.

### Integrity of the telomere capping complex in *m-tefu^5190^* embryos

The uncapping phenotype in *m-tefu^5190^* embryos suggests defects in telomere capping complexes. We along with others have shown that HOAP and HipHop are constitutive components of a telomere capping complex (Rashkova *et al.* 2002; Cenci *et al.* 2003; Gao *et al.* 2010b). We also showed that loss of ATM function does not prevent the telomeric binding of HOAP, nor does it affect its steady-state level in larval neuroblasts (Bi *et al.* 2004; Rong 2008b; Gao *et al.* 2010b). In contrast, we observed a reduction of loading of Hiphop to telomeres in the *tefu^stg^* mutants, accompanied by a significant drop in HipHop level (Gao *et al.* 2010b).

In *m-tefu^5190^* embryos, similar to *atm*-null larvae, we did not observe a reduction of HOAP by Western blot (Figure 3C). However, we did detect a significant reduction in HipHop level in *m-tefu^5190^* embryos (Figure 3C), again consistent with results from *atm*-null larvae. Therefore, loss of ATM function consistently reduces the abundance of HipHop protein, possibly because inefficient loading of HipHop to telomeres leads to its destabilization. This proposition is consistent with our immunostaining results that show the lack of HipHop signal on telomeres in most *m-tefu^5190^* nuclei (Figure 3D). However, we did observe HipHop foci in some nuclei (Figure 3D), suggesting that the effect of loss of ATM on HipHop loading is partial, similar to the situation in larval neuroblasts.

One of the most interesting aspects of this study is our discovery that cells from different stages of development can react very differently to the same genetic mutation. We envision that HipHop needs to be loaded onto newly replicated telomeres for their protection, and this loading requires the function of ATM. Perhaps, as we and others have proposed, ATM is essential for telomeric processing. Given the speed of the cell cycle (10−20 min) in early embryos, efficient telomere processing would be more stringently required, such that even a partial loss of ATM could have a strong effect over a few divisions. On the contrary, cell cycles in somatic tissues are much longer, such that a partial loss of function might be much better tolerated.

### DNA damage induced kinase activity is normal in *tefu^ZIII-5190^* mutants

**Figure 4:**
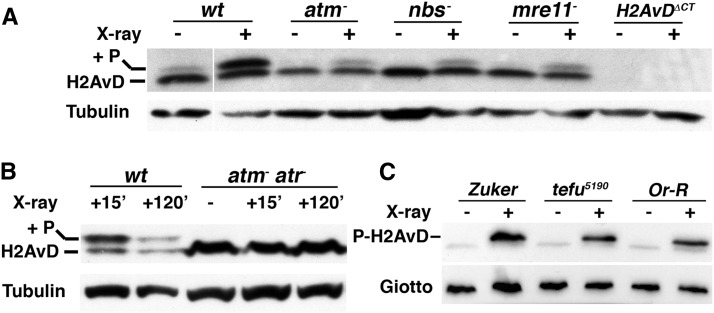
Damage-induced H2AvD phosphorylation is normal in *tefu^ZIII-5190^*. (A) Dependence of H2AvD phosphorylation on ATM and MRN. Extracts of the indicated genotypes were made from proliferating tissues in third instar larvae before (−) or 15 min after (+) irradiation. Membranes were probed with an antibody that recognizes both the phosphorylated (+P) and the unphosphorylated forms of H2AvD. For flies with the *H2AvD^ΔCT^* genotype, the only functional *H2AvD* copy has a C-terminal truncation that deletes the antibody epitope, and serves as a negative control. Tubulin was used as a loading control. (B). H2AvD phosphorylation activity in *atm atr* double mutant larvae. Extracts were taken from animals before (−), 15′ after, or 120′ after irradiation of either *wt* or *atm atr* double mutant larvae. (C) H2AvD phosphorylation in the *tefu^ZIII-5190^* mutant. Membranes were probed with an antibody specifically recognizes P-H2AvD. Two wild-type controls were included: Or-R and Zuker, which is the parental stock for *tefu^ZIII-5190^*. The Giotto protein was used as a loading control.

ATM is a protein kinase, and its kinase activity is critical for telomere maintenance in yeast (Mallory and Petes 2000). To investigate whether the reduction of kinase activity is the underlying defect in *tefu^ZIII-5190^* mutants, we used DNA damage-induced phosphorylation of the H2AX variant (H2AvD in *Drosophila*, Madigan *et al.* 2002) as an *in vivo* readout for ATM kinase activity. It has been previously shown that ATM is important for H2AvD phosphorylation induced by DNA damage (Joyce *et al.* 2011). Using proliferating cells from third instar larvae, we found that H2AvD phosphorylation (P-H2AvD) largely depends on ATM and the MRN complex, as greatly reduced levels of P-H2AvD induced by X-ray irradiation were observed in single mutants of these genes (Figure 4A). In addition, we found that most if not all of the H2AvD phosphorylation activities can be attributed to the ATM and its related ATR kinases, as a double mutant essentially abolishes P-H2AvD (Figure 4B).

When *tefu^ZIII-5190^* mutant larvae were irradiated, we observed a robust H2AvD phosphorylation, similar to the response from wild-type cells (Figure 4C). This result suggests that ATM encoded by the *tefu^ZIII-5190^* allele retains its ability to modify H2AvD upon DNA damage, and that loss of kinase activity might not be the underlying cause for telomere uncapping in *m-tefu^5190^* embryos.

We considered measuring P-H2AvD in embryos since the terminal phenotype of *tefu^ZIII-5190^* is embryonic lethal. However, damage-induced P-H2AvD happens on chromatin, and the high maternal deposition of free histones would make the results difficult to interpret. In addition, *m-tefu^5190^* embryos likely experience DNA damage due to telomere instability, which could further complicate the situation.

We don’t believe that the seemingly normal P-H2AvD level induced by X-ray is due to a preponderance of the maternal ATM in *tefu^ZIII-5190^* larvae. Using telomere fusion as an indicator, we found that the maternal ATM function is lost before the third instar stage. To estimate the timing of the loss of ATM function during development, we took advantage of the fact that telomere fusions in the *tefu^stg^* mutant result in chromosome bridges during mitosis (Bi *et al.* 2004). These bridges can be detected by staining mitotic chromatin for phosporylated histone H3. We staged homozygous mutants as first, second, and third instar larvae. We detected no mitotic bridges in more than 200 nuclei from 50 first instar larvae, but discovered bridges in 16.1% of the mitotic nuclei (n = 1043) from eight second instar animals, which is similar to the frequency from late third instar *tefu^stg^* larvae (20%, Bi *et al.* 2004). This led us to conclude that the loss of maternal ATM function likely occurs between the first and second instar stages.

H2AvD phosphorylation is severely compromised in *tefu* loss of function mutants, yet a normal kinase function in *tefu^ZIII-5190^* does not prevent telomere fusion. To reconcile these observations, we suggest that the damage-induced kinase activation of ATM is distinct from its kinase function at telomeres, possibly due to different ATM targets at telomeres *vs.* damage sites. If true, this predicts that the N-terminal portion of ATM deleted in *tefu^ZIII-5190^* might be responsible for interacting with a telomere-specific ATM target.

Here we have characterized a hypomorphic mutation in the conserved ATM checkpoint protein in *Drosophila* that specifically disrupts telomere capping during early embryonic cell divisions. The mitotic segregation defects are very similar to those observed in embryos that are genetically devoid of maternal ATM (Silva *et al.* 2004).

One long-standing question regarding ATM function in telomere maintenance concerns the identity of the targets of its kinase activity. Recently, the Ccq1 protein has been identified as an ATM target that is important for telomere protection in fission yeast (Moser *et al.* 2011). The availability of a maternal lethal *atm* mutation could facilitate the identification of similar targets in *Drosophila*. First, a large amount of mutant embryos is easy to collect and makes biochemical purification an attractive approach. Second, the synchrony of the early cell cycles simplifies both cytological and molecular characterizations. Finally, the hypomorphic nature of the *tefu^ZIII-5190^* allele should permit screening of enhancer/suppressor mutations, facilitating the genetic identification of new members of the ATM pathway.

## Supplementary Material

Supporting Information
