# Supplementary material for: A Hypomorphic Mutation Reveals a Stringent Requirement for the ATM Checkpoint Protein in Telomere Protection During Early Cell Division in Drosophila
Source: G3 (Bethesda). 2013 Jun 1;3(6):1043–8. doi: 10.1534/g3.113.006312 (PMC3689801; doi:10.1534/g3.113.006312)
Supplement: Supporting Information [file supp_3_6_1043__index.html]

A Hypomorphic Mutation Reveals a Stringent Requirement for the ATM Checkpoint Protein in Telomere Protection During Early Cell Division in Drosophila — Supporting Information 

# A Hypomorphic Mutation Reveals a Stringent Requirement for the ATM Checkpoint Protein in Telomere Protection During Early Cell Division in *Drosophila*

## Supporting Information for Morciano *et al.*, 2013

**Files in this Data Supplement:**

- Table S1 - Primer list (PDF, 121 KB)
