# Supplementary material for: A Hypomorphic Mutation Reveals a Stringent Requirement for the ATM Checkpoint Protein in Telomere Protection During Early Cell Division in Drosophila
Source: G3 (Bethesda). 2013 Jun 1;3(6):1043–8. doi: 10.1534/g3.113.006312 (PMC3689801; doi:10.1534/g3.113.006312)
Supplement: Supporting Information [file supp_g3.113.006312_TableS1.pdf]

G3 · Genes | Genomes | Genetics

A hypomorphic mutation reveals a stringent requirement for the ATM checkpoint protein in telomere protection during early cell division in *Drosophila*

Patrizia Morciano[1,2], Yi Zhang[1], Giovanni Cenci[2], Yikang S. Rong[1,3]

1: LBMB, NCI, NIH, USA
2: SAPIENZA, University of Rome, Rome, Italy
3: Corresponding Author: rongy@mail.nih.gov

DOI: 10.1534/g3.113.006312

Table S1 Primer list

Name	Sequence (5' to 3')	Combination
For identification of tefu mutation		
1	CACAACAACTTCGGGTTTCGA	1+2
2	ATGTGCGTATGCTCTGATGG	
3	GCATCCTGTCGAAGATCATC	3+4
4	TGAGTTTGTCTACTGCGTCC	
5	CACTGTCCAGTCGACAGTATG	5+6
6	GTCGCAGAATGAGTGAGTTC	
7	GTCGCAGAATGAGTGAGTTC	7+8
8	CTGCGAATACATACGAATC	
9	CAGTGATTGCTAGCCATAACC	9+10
10	TTGGAAGAGACAACGGACTG	
11	TGGCGTCGGACTTAACAGAAATG	11+12
12	CGCAAAACACACGCCAGTG	
13	CGCTGATGGATTTACTGCAC	13+14
14	TTGTTTGATTCAATCCAATTA	
15	CAAAAGCCTTTCCATTGTGCAGATC	15+16
16	TACTTCAGCTGAGTAGCGTG	
17	GAAGATCTCTCGAATCGTCG	17+18
18	CCAGAATACTGAAATGCATC	
19	CGCTACTCAGCTGAAGTATC	19+20
20	CTGGAGGATGTCAACGAG	
21	ACGTAAAGTCATAAGACCAAG	21+22
22	TTCTAGTTCTCCATCGGCAC	
23	GTTCGAGAAACCTTGCCTTC	23+24
24	GAGAGCTTTTCATCCAGCTG	
25	CATGGATGTCTTACAGGCCG	25+26
26	CGAGCTTTGCTTCTTGTGCA	
For telomere fusion isolation		
1. HeT-A453rev	ACTTCCCATTGCATCGCTCGTT	1+4
2. HeT-A1196rev	GGCGGAAAAATGCTGGGAGTTAC	2+4
3. HeT-A1751rev	CGCATGGGGCCACCTGTAG	3+4
4. HeT-A1997rev	GTGGCGGGGGTGGTTCTTG	
5. HeT-A2615rev	ATTTTTGGCGATGGCGTGGTA	3+5
